# Effects of Dietary Protein Levels on Growth, Serum Physiology, Protein and Lipid Metabolism, and Antioxidant Responses in Black Carp (*Mylopharyngodon piceus*)

**DOI:** 10.3390/metabo16060391

**Published:** 2026-06-04

**Authors:** Jinjing Zhang, Songting Yang, Yukai Zhu, Jiaxing Yu, Yuanyuan Zhang, Jie Li, Chengye Lin, Chenglong Wu

**Affiliations:** National-Local Joint Engineering Laboratory of Aquatic Animal Genetic Breeding and Nutrition (Zhejiang), Huzhou Normal University, 759 East 2nd Road, Huzhou 313000, China; zjj18388550747@outlook.com (J.Z.); yangsongting1@outlook.com (S.Y.); zyk20220516@outlook.com (Y.Z.); y15615528313@outlook.com (J.Y.); y2424507372@outlook.com (Y.Z.); lijie158784@outlook.com (J.L.); linjiangaichitang@outlook.com (C.L.)

**Keywords:** black carp, digestive enzymes, gene expression, antioxidant homeostasis, histology

## Abstract

**Background**: Dietary protein optimization is an important nutritional strategy for improving growth and physiological responses, and antioxidant homeostasis in fish. **Methods**: In this study, 540 black carp (initial body weight: 10.50 ± 1.00 g) were randomly assigned into recirculating tanks (500 L) fed with six dietary protein levels (30–44% crude protein) for an 8-week feeding trial with triplicates per treatment and 30 fish per replicate. After the trial, fish body, blood, hepatopancreas, and intestinal samples were collected for body composition, serum biochemical parameters, metabolism, and antioxidant indices’ analyses. **Results**: Results showed fish fed 38% protein (PT38) exhibited the highest weight gain (*p* < 0.05), with no further improvement at higher protein levels. Compared with PT30 group, PT38 group significantly promoted protein deposition by upregulating transcript levels of insulin-like growth factors (*IGFs*) via activating mechanistic target of rapamycin (*mTOR*) signaling pathway. PT38 could improve fatty acid oxidation by heightening levels of carnitine palmitoyl transferase 1α (*CPT1α*), peroxisome proliferator-activated receptor α (*PPARα*) and *PPARδ*. Meanwhile, PT38-PT41 significantly inhibit expression of fatty acid synthesis and lipid droplet deposition-related genes, including acetyl-CoA carboxylase (*ACC*), fatty acid synthase (*FAS*), and perilipin 2 (*p* < 0.05). PT38 significantly enhanced antioxidant homeostasis by increasing levels of superoxide dismutase (SOD), catalase (CAT), and glutathione peroxidase (GPx) via activating nuclear factor erythroid 2-related factor 2 (Nrf2) signaling pathway. **Conclusions**: Overall, Under the current experimental conditions, 38% dietary protein is suitable for promoting growth performance, improving protein and lipid metabolism, and enhancing antioxidant homeostasis in black carp.

## 1. Introduction

Protein is a key nutrient for organisms, significantly influencing metabolic and immune functions in humans, animals, and fish [1]. Previous studies have shown that an appropriate dietary protein level can effectively promote normal growth and nutrient metabolism while enhancing antioxidant capacity and immune function [2,3,4]. However, insufficient dietary protein generally suppresses growth performance and feed efficiency and may disturb lipid metabolism and promote fat deposition [5,6]. Excessive dietary protein not only induces metabolic disorders but also heightens nitrogen excretion, elevates feed costs, and subsequently causes rearing environmental pollution [7,8]. In addition, high dietary protein intake can increase xanthine oxidase activities, cause oxidative stress, and deteriorate gut health in cultured animals [9,10]. Therefore, defining suitable dietary protein requirements is crucial for balancing growth, immune regulation, and environmental protection in cultured animal species.

Dietary protein plays an essential role in regulating protein accretion and lipid metabolic homeostasis [11,12]. Protein synthesis, a key process underlying normal growth responses, is primarily regulated by the mechanistic target of rapamycin (mTOR) signaling pathway, especially through downstream effectors such as S6 kinase 1 (S6K1) and eukaryotic translation initiation factor 4E-binding protein (4E-BP) [13,14]. Dietary protein deficiency can downregulate the expression levels of *TOR* and *S6K1* and consequently impair protein synthesis [15,16]. Growth-related endocrine factors, such as insulin-like growth factor I (IGF-I) and insulin-like growth factor II (IGF-II), may also respond to dietary protein availability and interact with these pathways to regulate cell growth and development [13,17,18]. These studies have noted the necessity of determining how dietary protein levels modulate the TOR signaling pathway, combined with endocrine factors, in different animal species. In addition, numerous studies of animals have found that different levels of dietary protein can regulate body lipid contents by altering lipolysis, lipogenesis, and lipid transport [19,20]. These lipid metabolism processes are mainly regulated by upstream key regulating signal molecules, such as peroxisome proliferator-activated receptors (PPARs) and sterol regulatory element-binding proteins (SREBPs) [21]. However, the effects of graded dietary protein levels on lipid metabolism in black carp remain insufficiently understood.

Dietary protein levels may also affect oxidative status [22]. In animals, changes in nutrient and energy metabolism can alter the production of reactive oxygen species (ROS), thereby influencing oxidative stress and antioxidant defense [23,24]. Antioxidant molecules and enzymes, such as reduced glutathione (GSH), superoxide dismutase (SOD), catalase (CAT), and glutathione peroxidase (GPx), play important roles in maintaining redox balance and are regulated by the nuclear factor erythroid 2-related factor 2/Kelch-like ECH-associated protein 1 (Nrf2/Keap1) signaling pathway [25,26]. Previous studies have found that low-protein diets may weaken antioxidant capacity, whereas appropriate protein intake can enhance antioxidant enzyme activities and improve physiological status [22,27]. Therefore, elucidating the regulatory effects of different dietary protein levels on redox homeostasis and antioxidant defense mechanisms in cultured freshwater fish species is of great significance for evaluating their nutritional requirements and health status.

Black carp (*Mylopharyngodon piceus*) is a carnivorous fish species (http://www.fishbase.org, accessed on 11 April 2026) characterized by rapid growth, high yield, and favorable flesh quality [28]. According to data from the China Fisheries Statistical Yearbook, national black carp production in China reached 853,498 tons in 2024 [29]. With the rapid development of black carp culture, the associated feed-processing industry has also expanded markedly with the increasingly widespread use of formulated feed and related technology. Recent research has primarily concentrated on the effects of amino acids [30], vitamins [31,32], and minerals [33] on the growth performance and metabolism of black carp. Nevertheless, studies on black carp regarding the systematic relationship between different levels of dietary protein and serum physiology, growth, endocrine functions, antioxidants, and immune responses are still limited. Therefore, we hypothesized that dietary protein levels would significantly affect growth performance, nutrient metabolism, antioxidant responses in juvenile black carp, and that an appropriate dietary protein level would improve growth and physiological homeostasis. Based on this hypothesis, the present study examined the impacts of varying dietary protein levels on the growth performance and metabolic responses in juvenile black carp, aiming to identify the suitable protein requirement and provide a theoretical basis for feed formulation.

## 2. Materials and Methods

### 2.1. Diet Formulation

Six diets with graded protein levels were formulated containing protein concentrations of 30% (PT30), 33% (PT33), 36% (PT36), 38% (PT38), 41% (PT41), and 44% (PT44), with corresponding measured protein levels of 30.56%, 33.54%, 36.13%, 38.35%, 41.41%, and 43.88%, respectively (Table 1). Feed preparation was performed based on a previously described protocol [34], in which ingredients were progressively adjusted according to the formulation scheme and ground to pass through a 60-mesh sieve. Pellets (2.00 mm diameter) were manufactured using a twin-screw extrusion system, oven-dried at 40 °C with continuous airflow, and stored for subsequent use. The final amino acid (AA) composition was quantified on a dry-weight basis using an AA analyzer (L-8900, Hitachi High-Technologies, Tokyo, Japan) (Table 2).

### 2.2. Feeding Trial

All experimental procedures were reviewed and approved by the Ethics Committee of Huzhou University (Huzhou, China), and complied with the relevant regulations regarding the care and use of experimental animals. Black carp juveniles with mixed-sex population were obtained from Deqing Biotechnology Co., Ltd. (Huzhou, Zhejiang, China). Before the formal experiment, the fish were acclimated for 1 week using the control diet (PT30). After acclimation, 540 fish with similar body lengths were randomly selected and individually weighed to ensure the uniformity of initial body weight, with an average initial body weight of 10.50 ± 1.00 g. The fish were then randomly distributed into 18 recirculating aquaculture tanks (500 L each), with 30 fish stocked per tank. During the experimental period, fish were hand-fed to apparent satiation three times daily at 08:00, 12:00, and 17:00. The feeding amount was adjusted weekly according to feeding response and growth performance. One hour after each feeding, uneaten feed in each tank was collected by siphoning, dried in a forced-air oven at 70 °C to constant weight, and weighed to calculate feed intake and feed conversion ratio (FCR). During the trial, the pH was 6.9–7.1, and dissolved oxygen was at least 5.80 mg/L. A natural photoperiod was maintained for lighting, and water temperature was maintained at 26–28 °C, as previously described [35].

### 2.3. Sample Collection

Before sampling, the fish were fasted for 24 h. They were then anesthetized with 100 mg/L MS-222 (Sigma, St. Louis, MO, USA). The number of surviving fish in each tank was recorded, and body length and body weight were measured to calculate growth performance indices. Following the method described by Zhang et al. [36], 15 fish were randomly selected from each tank, and blood samples were collected from the caudal vein. Of the collected blood, 0.3 mL of whole blood was transferred into heparinized Eppendorf tubes for hematological analysis. The remaining blood samples were kept at 4 °C for 24 h and then centrifuged at 3000× *g* for 10 min. The supernatant serum was collected and stored at −80 °C until further analysis. Following the procedure described by Jia et al. [32], the fish were rapidly dissected on ice after blood collection. The viscera were removed and weighed, and the hepatopancreas and intestine were separated, with 15 samples collected for each tissue. The hepatopancreas was also weighed to calculate the VSI and HSI. The isolated hepatopancreas, intestine, and dorsal muscle samples were immediately frozen in liquid nitrogen and then stored at −80 °C for subsequent analyses. After sampling, three fish were randomly selected from each tank and stored at −20 °C for subsequent proximate composition analysis.

### 2.4. Proximate Composition

Sample analyses were conducted following standard procedures established by the AOAC [37]. Moisture levels in the experimental diets were measured using oven drying at 105 °C until constant weight, whereas moisture content in whole-fish tissues was measured using a freeze dryer (Alpha2-4 LSC Basic, Martin Christ Gefriertrocknungsanlagen GmbH, Osterode am Harz, Germany). To determine crude protein, an automated Dumas nitrogen analyzer (Rapid N exceed, Elementar Analysensysteme GmbH, Frankfurt, Germany) was employed, while Soxhlet extraction and 550 °C muffle furnace incineration were used to measure crude lipid and ash, respectively.

### 2.5. Biochemical Analyses

An automatic biochemical analyzer (LW C400; Shenzhen Landwind Medical Instrument Co., Shenzhen, China) was employed to determine the serum biochemical indices. The measured parameters included aspartate aminotransferase (AST, AST01), total cholesterol (TC, CH01), triglyceride (TG, TG01), low-density lipoprotein cholesterol (LDL-C, LDL01), total bile acid (TBA, TBA01S), glucose (GLU, GLU01), alkaline phosphatase (ALP, ALP01), albumin (ALB, ALB01), alanine aminotransferase (ALT, ALT01), high-density lipoprotein cholesterol (HDL-C, HL01), and blood urea nitrogen (BUN, URE01). Serum measurements were performed in at least 3 replicates.

### 2.6. Determination of Digestive Enzyme and Antioxidant Indicators

Hepatopancreas and intestinal tissues were pulverized into fine powder in liquid nitrogen and then homogenized. Centrifugation was then conducted at 3000× *g* for 15 min at 4 °C, and the supernatants were harvested and retained for additional analysis. Commercial assay kits (Jiancheng Bioengineering, Nanjing, China) were employed to determine the following parameters: trypsin (TRY, A080-2-2), T-SOD (A001-1-2), glutathione s-transferase (GST, A004-1-1), GSH (A006-2-1), TG (A110-1-1), total antioxidant capacity (T-AOC, A015-2-1), amylase (AMS, C016-1-1), CAT (A007-2-1), MDA (A003-1-2), GPx (A005-1-2), and glutathione reductase (GR, A062-1-1). In addition, lipopolysaccharide (LPS, A054-1-1) and FAS (HB207-QT) were measured using fish-specific commercial kits (Shanghai Hengyuan Biotech, Shanghai, China). Each assay was performed with three replicates.

### 2.7. Oil Red O Staining and Transmission Electron Microscopy

Hepatopancreas tissues (0.5 cm × 0.5 cm × 0.5 cm) were gently rinsed with 0.6% saline and embedded in OCT compound for frozen section preparation. The frozen sections were then subjected to Oil Red O (ORO) staining. Then representative histological images were recorded using a digital camera [34]. Quantitative image was firstly selected from five regions (upper, lower, left, right, and center) in each hepatopancreas section and performed with 400× magnification using the K-Viewer software (version 1.9; accessed on 16 January 2025) (https://kv.kintoneapp.com/en/user/). Representative images were selected from comparable tissue regions under the same microscopic magnification and imaging conditions. Tissue folds, damaged areas, large vessels, and blank regions were avoided during field selection. For each treatment, three biological replicates were examined, and multiple non-overlapping fields were observed for each sample. Image observation and representative image selection were performed in a blinded manner. The relative area of lipid droplet accumulation and numbers of lipid droplet were quantified using ImageJ software (version 1.53a, National Institutes of Health, Bethesda, MD, USA) according to the method supplied by Mehlem et al. [38].

After incubation in OsO_4_ diluted in 0.1 M PB (pH 7.4) for 1–2 h, dehydration was carried out through a graded ethanol series (30–100%). Hepatopancreas samples were infiltrated with a mixture of acetone and Embed 812 (1:1 for 2–4 h and 1:2 overnight) and then embedded in Embed 812 and polymerized at 65 °C for 48 h. Sections (60–80 nm) were cut and mounted on copper grids, followed by contrasting with uranyl acetate and lead citrate [39]. Sections were observed using a transmission electron microscope (TEM) (JEM-1400 Flash, JEOL, Tokyo, Japan). Each assay was performed with three replicates.

### 2.8. Quantification of Gene Expression

Total RNA was isolated from hepatopancreas samples using TRIzol reagent according to the manufacturer’s instructions (Invitrogen, Waltham, MA, USA). Before cDNA synthesis, RNA concentration and purity were assessed using a Nanodrop spectrophotometer, and RNA integrity was evaluated by agarose gel electrophoresis. Subsequently, the extracted RNA was reverse-transcribed into cDNA using the MonScript RT-PCR Kit following the protocol provided by Monad Biotech (Wuhan, China). All cDNA samples obtained from different treatment groups were stored at −80 °C until subsequent qPCR analysis. Gene-specific primers were designed using Primer 5.0 software, and detailed gene information is provided in the Supplementary Materials. All primers were synthesized by Biosune Biotech Co., Ltd. (Shanghai, China). β-actin was used as the internal reference gene. qPCR analysis was performed using SYBR Green Real-time PCR Master Mix (Takara, Beijing, China) on a CFX96 real-time PCR detection system (Bio-Rad, Hercules, CA, USA). The experimental procedures followed the methods described by Wu et al. [30] and Jia et al. [32]. Relative gene expression levels were calculated using the 2^−ΔΔCT^ method. For each treatment group, three biological replicates were analyzed, with one sample representing one biological replicate, and each biological replicate being derived from an independent tank.

### 2.9. Statistical Analysis

Data on growth performance were calculated as follows:

Weight gain (WG, %) = 100 × (final body weight − initial body weight)/initial body weight

Specific growth rate (SGR, %/day) = 100 × [ln (final body weight) − ln (initial body weight)]/feeding period (day)

Viscerosomatic index (VSI, %) = viscera weight/final body weight × 100

Hepatosomatic index (HSI, %) = hepatopancreas weight/final body weight × 100

Intestinal somatic index (ISI, %) = intestinal weight/final body weight × 100

Condition factor (CF, g/cm^3^) = final body weight/fish body length^3^ × 100

Feed conversion ratio (FCR) = dry weight of the feed intake/(final body weight-initial body weight)

All experimental data are expressed as mean ± SD. Prior to statistical analysis, data were tested for normality using the Shapiro–Wilk test and for homogeneity of variance using Levene’s test. Fold-change values for gene expression were log-transformed before analysis. One-way analysis of variance (one-way ANOVA) was performed using SPSS 27.0 to evaluate the effects of different dietary protein levels on the measured parameters. When significant differences among treatments were detected, Duncan’s multiple range test was applied for post hoc comparisons. Duncan’s test was selected based on its widespread use in aquaculture and animal nutrition studies, as well as its relatively high sensitivity for detecting differences between multiple treatment groups. Statistical significance was set at *p* < 0.05. Given the number of response variables analyzed, results were interpreted based on statistical significance and biological relevance.

## 3. Results

### 3.1. Growth Performance

As dietary protein levels increased, FBW, WG, and SGR in black carp increased initially and then leveled off (Table 3). Fish in the PT38 group showed higher FBW, WG, and SGR than those in the PT30 and PT33 groups (*p* < 0.05), whereas no differences were detected between the PT36–PT44 groups (*p* > 0.05). By contrast, FCR gradually declined with increasing dietary protein level and reached its lowest value in the PT44 group (*p* < 0.05). VSI, HSI, and ISI generally decreased as dietary protein level increased and then remained relatively stable. The PT30 group exhibited higher VSI and HSI values than the PT36–PT44 groups (*p* < 0.05), with values comparable with those of the PT33 group (*p* > 0.05). The ISI value in the PT44 group was lower than in the PT30–PT38 groups (*p* < 0.05), whereas CF did not vary between treatments (*p* > 0.05). With increasing dietary protein, whole-body crude protein and crude ash contents gradually increased, whereas crude lipid content showed the opposite pattern. Compared with the PT30 group, the PT38 group had elevated crude protein and crude ash contents (*p* < 0.05), after which these parameters remained largely unchanged. The PT44 group showed the lowest crude lipid content, although no difference was observed between the PT38–PT44 groups (*p* > 0.05).

### 3.2. Serum Biochemical Parameters

With increasing dietary protein levels, serum HDL-C, TG, BUN, LDL-C, and TC generally declined (Table 4). Fish in the PT44 group showed lower concentrations of HDL-C, LDL-C, TG, and TC than those in the PT30 group (*p* < 0.05). By contrast, ALP and ALB were elevated in the PT38 group relative to the PT30 group (*p* < 0.05). TBA showed a rise followed by a decline as dietary protein increased, and its level in the PT44 group was lower than in the PT33 group (*p* < 0.05). Moreover, AST and GLU increased with increasing dietary protein levels and then tended to stabilize. AST and GLU in the PT41 group exceeded those in the PT30 group (*p* < 0.05), whereas comparable values were observed between the PT41 and PT44 groups (*p* > 0.05). By contrast, ALT activity remained unchanged among all dietary treatments (*p* > 0.05).

### 3.3. Activities of Digestive Enzymes in the Hepatopancreas and Intestine

Table 5 shows that TRY and LPS activities in the hepatopancreas and intestine rose with increasing dietary protein, reaching maximal values in the PT38 group and subsequently declining at higher dietary protein levels (*p* < 0.05). Intestinal LPS activity remained similar among PT36–PT41 (*p* > 0.05). In the hepatopancreas, AMS activity decreased as dietary protein increased, with the lowest value observed in PT44 compared with PT38 (*p* < 0.05). By contrast, intestinal AMS displayed a rise-then-fall pattern, reaching a higher level in PT38 than in PT30 (*p* < 0.05).

### 3.4. Gene Expression of Protein Synthesis-Related Genes in the Hepatopancreas

**Figure 1:**
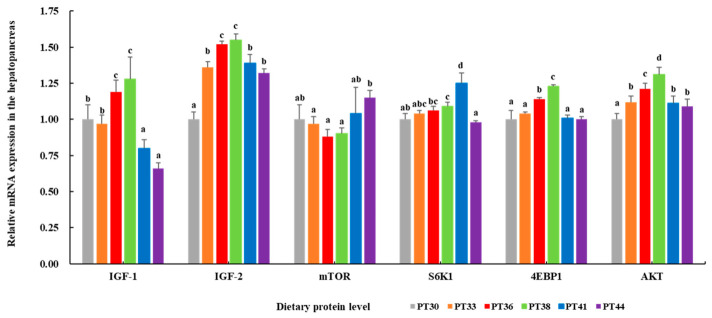
Effects of dietary protein levels on the relative mRNA expression of genes involved in protein synthesis regulation in the hepatopancreas of black carp (*Mylopharyngodon piceus*). Data are expressed as mean ± SD (n = 3 biological replicates per treatment). Different lowercase letters above the bars indicate significant differences among groups according to one-way ANOVA followed by Duncan’s multiple range test (*p* < 0.05); bars without lowercase letters indicate no significant differences among groups. PT30, PT33, PT36, PT38, PT41, and PT44 represent the dietary protein levels of 30%, 33%, 36%, 38%, 41%, and 44%, respectively.

The transcript levels of *IGF-1*, *IGF-2*, *S6K1*, *4EBP1*, and *AKT* increased and then decreased with further elevation of dietary protein levels (Figure 1). Compared with the PT30 group, fish in the PT38 group showed higher *IGF-1*, *IGF-2*, *4EBP1*, and *AKT* expression (*p* < 0.05). For *mTOR* expression levels, the PT44 group was significantly higher than the PT33, PT36, and PT38 groups (*p* < 0.05), and *IGF-1* and *IGF-2* also showed comparable levels between the PT36 and PT38 groups (*p* > 0.05). Meanwhile, *S6K1* expression in the PT41 group exceeded that in the PT30 group (*p* < 0.05).

### 3.5. Hepatopancreatic Gene Expression Involved in Lipid Metabolism

**Figure 2:**
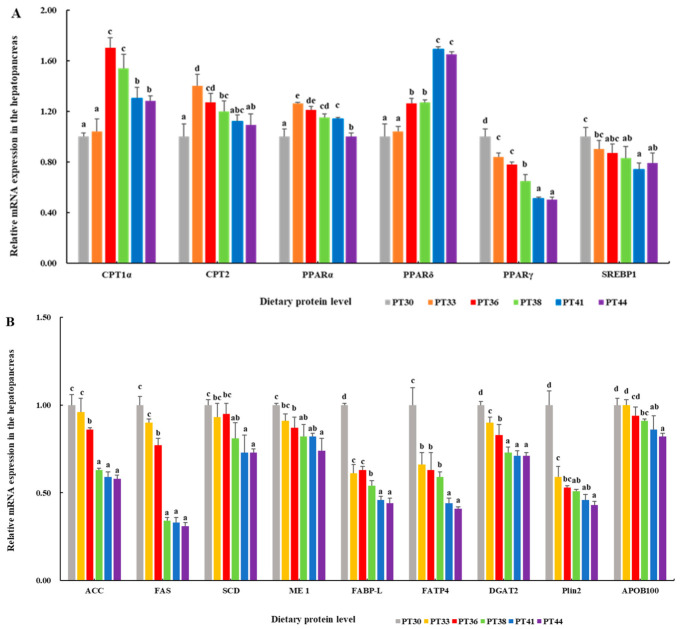
Effects of dietary protein levels on the relative mRNA expression of genes involved in fatty acid oxidation and transcriptional regulation (**A**), and lipogenesis and lipid transport metabolism (**B**), in the hepatopancreas of black carp (*Mylopharyngodon piceus*). Data are expressed as mean ± SD (n = 3 biological replicates per treatment). Different lowercase letters above the bars indicate significant differences among groups according to one-way ANOVA followed by Duncan’s multiple range test (*p* < 0.05); bars without lowercase letters indicate no significant differences among groups. PT30, PT33, PT36, PT38, PT41, and PT44 represent the dietary protein levels of 30%, 33%, 36%, 38%, 41%, and 44%, respectively.

As dietary protein level increased, the transcript levels of carnitine palmitoyl transferase 1 alpha (*CPT1α*), carnitine palmitoyl transferase 2 (*CPT2*), and *PPARα* showed an initial increase followed by a decline (Figure 2A). Of these, *CPT1α* and *CPT2* expression levels were higher in the PT36 and PT38 groups than in the PT30 group (*p* < 0.05). By contrast, the expression of *PPARγ* and *SREBP1* generally decreased with increasing dietary protein, and both genes showed lower expression in the PT44 group than in the PT30 group (*p* < 0.05). Meanwhile, *PPARδ* reached its highest expression level in the PT41 group (*p* < 0.05) but remained comparable with that in the PT44 group (*p* > 0.05).

Increasing dietary protein level also suppressed the transcription of genes involved in fatty acid synthesis in the hepatopancreas, including fatty acid-binding protein (*FABP-L*), acetyl-CoA carboxylase (*ACC*), fatty acid synthase (*FAS*), stearoyl-CoA desaturase (*SCD*), malic enzyme 1 (*ME1*), and fatty acid transport protein 4 (*FATP4*). Compared with the PT30 group, the expression levels of these genes were reduced in the PT38 and PT41 groups (*p* < 0.05). In addition, apolipoprotein B100 (*APOB100*), a key gene closely related to TAG transport, showed lower expression in the PT44 group than in the PT30 group (*p* < 0.05), while no difference was observed between the PT41 and PT44 groups (*p* > 0.05). Similarly, diacylglycerol O-acyltransferase 2 (*DGAT2*) and perilipin 2 (*Plin2*) were also downregulated in the PT44 group (*p* < 0.05), with comparable expression levels between the PT38–PT44 groups (*p* > 0.05) (Figure 2B).

### 3.6. Hepatopancreatic Gene Expression Involved in Cholesterol and Bile Acid Metabolism

**Figure 3:**
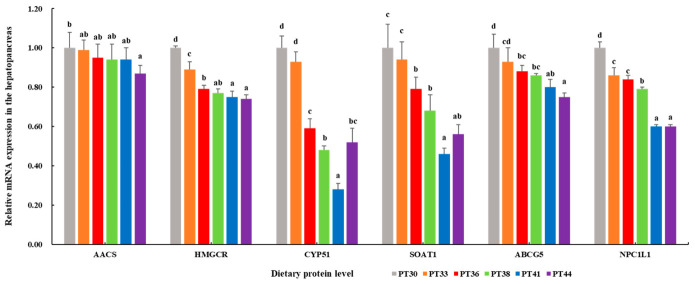
Effects of dietary protein levels on the relative mRNA expression of genes involved in cholesterol metabolism in the hepatopancreas of black carp (*Mylopharyngodon piceus*). Data are expressed as mean ± SD (n = 3 biological replicates per treatment). Different lowercase letters above the bars indicate significant differences among groups according to one-way ANOVA followed by Duncan’s multiple range test (*p* < 0.05); bars without lowercase letters indicate no significant differences among groups. PT30, PT33, PT36, PT38, PT41, and PT44 represent the dietary protein levels of 30%, 33%, 36%, 38%, 41%, and 44%, respectively.

As dietary protein level increased, the expression levels of genes involved in cholesterol metabolism in the hepatopancreas, including acetoacetyl-CoA synthetase (*AACS*), 3-hydroxy-3-methylglutaryl-CoA reductase(*HMGCR*), ATP-binding cassette subfamily g member 5 (*ABCG5*), and Niemann-pick c1-like 1 (*NPC1L1*), generally showed a downward trend (Figure 3), and their expression levels in the PT44 group were lower than those in the PT30 group (*p* < 0.05). By contrast, lanosterol 14α-demethylase (*CYP51*) and sterol O-acyltransferase 1 (*SOAT1*) displayed a pattern of initial decline followed by an increase. Compared with the PT30 group, the expression levels of *CYP51* and *SOAT1* were lower in the PT38 group (*p* < 0.05). Of the two, *SOAT1* showed no difference between the PT41 and PT44 groups (*p* > 0.05).

**Figure 4:**
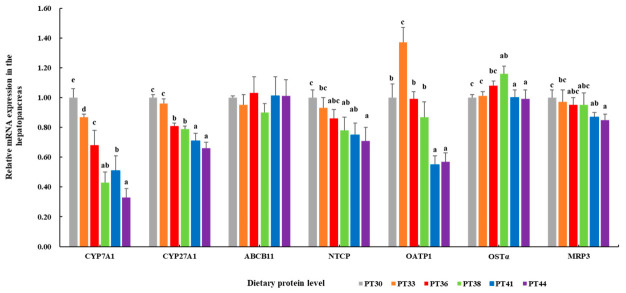
Effects of dietary protein levels on the relative mRNA expression of genes involved in bile acid metabolism in the hepatopancreas of black carp (*Mylopharyngodon piceus*). Data are expressed as mean ± SD (n = 3 biological replicates per treatment). Different lowercase letters above the bars indicate significant differences among groups according to one-way ANOVA followed by Duncan’s multiple range test (*p* < 0.05); bars without lowercase letters indicate no significant differences among groups. PT30, PT33, PT36, PT38, PT41, and PT44 represent the dietary protein levels of 30%, 33%, 36%, 38%, 41%, and 44%, respectively.

The transcript levels of genes related to bile acid synthesis, including Cholesterol 7α-hydroxylase (*CYP7A1*), sterol 27-hydroxylase (*CYP27A1*), Na^+^/Taurocholate transporting polypeptide (*NTCP*), and multidrug resistance-associated protein 3 (*MRP3*), gradually decreased with increasing dietary protein and were lower in the PT44 group than in the PT30 group (*p* < 0.05). Organic anion transporting polypeptide 1 (*OATP1*) and organic solute transporter alpha (*OSTα*) exhibited an initial increase followed by a decline and reached lower levels in the PT44 group (*p* < 0.05) while remaining comparable between the PT41 and PT44 groups (*p* > 0.05). By contrast, ATP-binding cassette subfamily B member 11 (*ABCB11*) expression remained stable across all dietary treatments, with no detectable difference between groups (*p* > 0.05) (Figure 4).

### 3.7. Antioxidant Responses in the Intestine and Hepatopancreas

As dietary protein increased, the T-SOD, CAT, GPX, and GSH activities/contents in the hepatopancreas and intestine generally exhibited a pattern of initial increase followed by a decline (Table 6). Compared with the PT30 group, SOD and GPX activities in the hepatopancreas, as well as GSH content in the intestine, were elevated in the PT38 and PT41 groups (*p* < 0.05). In particular, CAT activity and GSH content in the hepatopancreas, together with SOD, CAT, GPX, and T-AOC levels in the intestine, reached higher levels in the PT38 group (*p* < 0.05). By contrast, GST activity and MDA content in the hepatopancreas and intestine showed an opposite trend with increasing dietary protein, reaching lower levels in the PT44 group (*p* < 0.05). However, GST activity remained comparable between the PT38–PT44 groups (*p* > 0.05). In addition, T-AOC in the hepatopancreas declined in the PT41 group and then remained stable, whereas intestinal T-AOC peaked in the PT38 group (*p* < 0.05).

**Figure 5:**
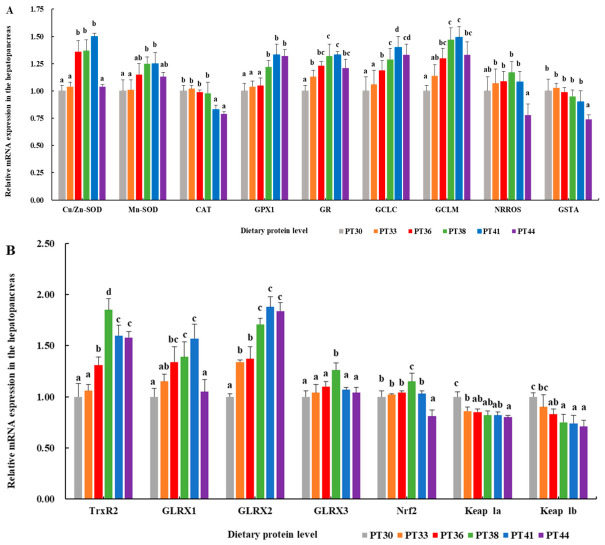
Effects of dietary protein levels on the relative mRNA expression of genes related to antioxidant defense in the hepatopancreas of black carp (*Mylopharyngodon piceus*). Antioxidant defense- and glutathione metabolism-related genes (**A**), and Nrf2/Keap1 signaling pathway- and redox regulation-related genes (**B**). Data are expressed as mean ± SD (n = 3 biological replicates per treatment). Different lowercase letters above the bars indicate significant differences among groups according to one-way ANOVA followed by Duncan’s multiple range test (*p* < 0.05); bars without lowercase letters indicate no significant differences among groups. PT30, PT33, PT36, PT38, PT41, and PT44 represent the dietary protein levels of 30%, 33%, 36%, 38%, 41%, and 44%, respectively.

With increasing dietary protein, the expression patterns of antioxidant-related genes in the hepatopancreas were generally consistent with the changes observed in antioxidant enzymes. The transcript levels of Cu/Zn superoxide dismutase (*Cu/Zn-SOD*), Manganese superoxide dismutase (*Mn-SOD*), *GPX1*, *GR*, glutamate-cysteine ligase catalytic subunit (*GCLC*), and glutamate-cysteine ligase modifier subunit (*GCLM*) increased initially and then declined (Figure 5A) and were higher in the PT38 and/or PT41 groups than in the PT30 group (*p* < 0.05). By contrast, CAT expression decreased in the PT41 group and then remained stable (*p* < 0.05). The expression of negative regulator of reactive oxygen species (*NRROS*) and glutathione s-transferase alpha (*GSTA*) gradually declined with increasing dietary protein level and reached lower levels in the PT44 group (*p* < 0.05). Meanwhile, *Nrf2*, thioredoxin reductase 2 (*TrxR2*), glutaredoxin 1 (*GLRX1*), glutaredoxin 2 (*GLRX2*), and glutaredoxin 3 (*GLRX3*) were upregulated in the PT38 group (*p* < 0.05) (Figure 5B). Of these, *GLRX1* remained unchanged across the PT36–PT41 groups (*p* > 0.05), and *GLRX2* showed comparable levels between the PT38, PT41, and PT44 groups (*p* > 0.05). By contrast, *Keap1a* and *Keap1b* exhibited an opposite pattern, with lower transcript levels in the PT36 group (*p* < 0.05), whereas no difference was detected among the PT36–PT44 groups (*p* > 0.05).

### 3.8. Histological Analysis of Hepatopancreas

**Figure 6:**
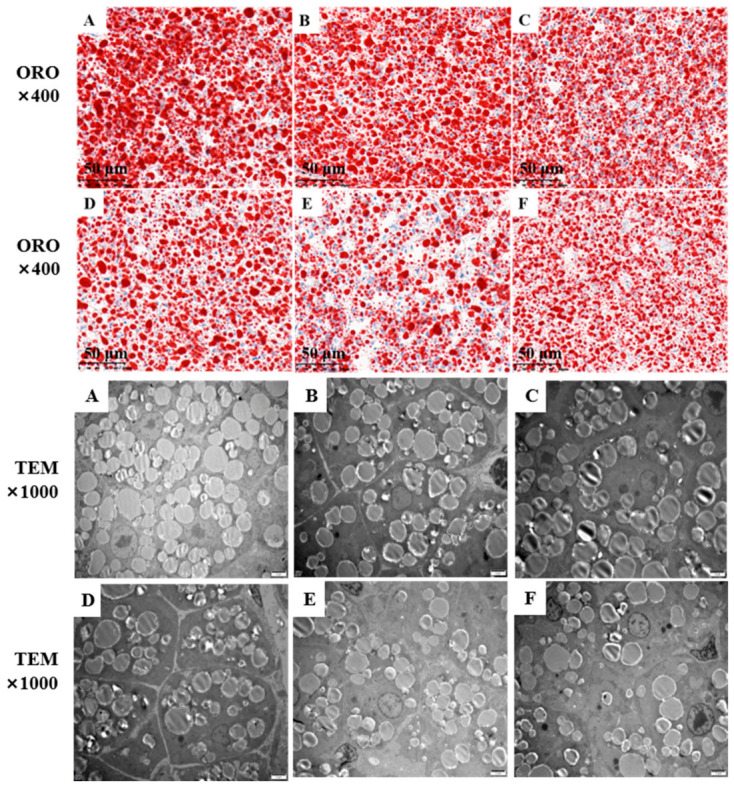
Oil Red O(ORO) staining and transmission electron microscopy (TEM) observations of hepatopancreas sections from black carp (*Mylopharyngodon piceus*) fed diets with different protein levels for 8 weeks. (**A**–**F**) represent the 30%, 33%, 36%, 38%, 41%, and 44% dietary protein groups, respectively, and each group includes both the Oil Red O-stained histological image and the corresponding electron microscopy image. Oil Red O-stained sections show lipid deposition in the hepatopancreas; transmission electron microscopy images show the number of lipid droplets in the hepatopancreas. Original magnification: Oil Red O staining, ×400; transmission electron microscopy, ×1000. Scale bars are indicated in the images.

**Figure 7:**
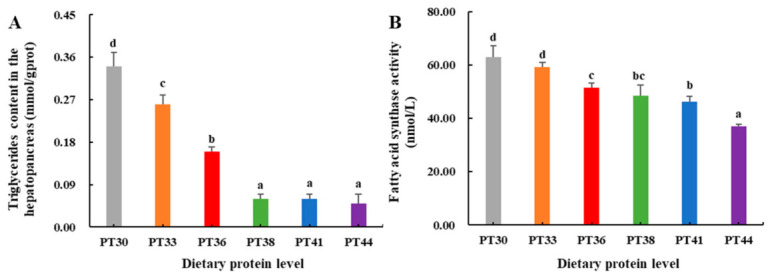
Effects of dietary protein levels on hepatopancreatic triglyceride content (**A**) and fatty acid synthase activity (**B**) in black carp (*Mylopharyngodon piceus*). Data are expressed as mean ± SD (n = 3 biological replicates per treatment). Different lowercase letters above the bars indicate significant differences among groups according to one-way ANOVA followed by Duncan’s multiple range test (*p* < 0.05); bars without lowercase letters indicate no significant differences among groups. PT30, PT33, PT36, PT38, PT41, and PT44 represent the dietary protein levels of 30%, 33%, 36%, 38%, 41%, and 44%, respectively.

With increasing dietary protein levels, evaluation of ORO-stained hepatopancreas sections revealed a significant reduction in lipid droplet accumulation, as indicated by the decreased red-stained areas. Compared with the PT30 group, lipid droplets were markedly reduced in the PT44 group. Consistently, TEM observations further confirmed abundant lipid droplets in PT30, whereas their abundance decreased noticeably in PT38–PT44 (Figure 6). In addition, hepatopancreatic TG content and FAS activity exhibited an inverse relationship with dietary protein level (Figure 7). Compared with PT30, TG content showed a decreasing tendency in PT38 (*p* < 0.05) and remained comparable between the subsequent treatments (*p* > 0.05). Meanwhile, compared with the PT30 group, FAS activity in the hepatopancreas was significantly decreased in the PT44 group (*p* < 0.05).

## 4. Discussion

Protein intake plays an essential role in supporting growth and development across humans and other animals [1]. In this study, WG and SGR peaked at PT38 and maintained stable levels with further increases in dietary protein content in black carp. These findings are consistent with those reported for crucian carp (*Carassius auratus*) [40] and striped catfish (*Pangasianodon hypophthalmus*) [41]. However, these values were lower than those reported for carnivorous and other omnivorous fish species, including sharpsnout seabream (*Diplodus puntazzo*) [42], largemouth bass (*Micropterus salmoides*) [43], and catfish (*Clarias magur*) [44], but higher than those in herbivorous fish, such as grass carp (*Ctenopharyngodon idella*) [45] and silver moony (*Monodactylus argenteus*) [46]. These differences may reflect species-specific variation. In addition, nutrient digestion and utilization largely depend on the activities of digestive enzymes that hydrolyze and facilitate the assimilation of feed components in the liver and intestine, thereby influencing growth performance [47]. Consistent with findings in red tilapia (*Oreochromis* spp.) [48] and yellow river carp (*Cyprinus carpio haematopterus*) [49], the present study showed black carp fed the 38% protein diet exhibited relatively high trypsin and lipase activities in the hepatopancreas and intestine, indicating that this protein level was more favorable for maintaining better digestive capacity. Although the feed conversion ratio was significantly reduced in the PT44 group, digestive enzyme activities did not continue to increase and instead showed a declining trend. This suggests that the improvement in feed utilization efficiency did not entirely depend on the sustained enhancement of digestive enzyme activity; instead, it may have been partly related to the increased substrate supply under the higher dietary protein level [50,51]. Meanwhile, no significant differences in the specific growth rate were observed between the PT36–PT41 groups, indicating that black carp growth had become relatively stable once dietary protein reached a certain range. Taken together, feed utilization efficiency, digestive enzyme activity, and growth performance did not change in complete synchrony. Overall, a dietary protein level of around 38% appears to be more appropriate for balancing digestive function and growth performance, whereas higher protein levels mainly contributed to a further improvement in feed utilization efficiency.

Furthermore, the whole-body crude protein proportion was elevated in the high-protein intake groups, similar to observations in peninsular carp (*Hypselobarbus pulchellus*) [52], suggesting that an adequate level of dietary protein can improve protein synthesis and deposition in black carp. Accumulating evidence indicates that the mTOR/4EBP signaling cascade is crucial for protein metabolic homeostasis and functions synergistically with the IGF system in controlling fish growth, development, and metabolic processes [17,53]. The significant upregulation of *IGF-1*, *IGF-2*, *4EBP1*, and *AKT* observed in the PT38 group suggests activation of the mTOR signaling pathway in the hepatopancreas of black carp, which may account for the enhanced protein deposition, consistent with observations in tilapia (GIFT: *Oreochromis niloticus*) [17] and abalone (*Haliotis discus hannai*) [54]. ALT and AST function are essential enzymes in the metabolic pathways of amino acids [55,56], and elevated serum ALT and AST levels are generally related to hepatic health status through the modulation of amino acid catabolism for energy production during excessive protein intake, starvation, nutrition imbalance, and other environmental stresses [57]. In this investigation, AST levels were increased in the high-protein group (PT44), whereas serum ALT levels did not differ significantly among the six groups, indicating that higher dietary protein intake can induce hepatic metabolic stress [57]. BUN in serum is an indispensable indicator of protein synthesis and utilization, and elevated levels suggest amino acid imbalance and inefficient protein utilization [58]. Serum BUN levels are positively correlated with dietary protein levels, as reported in the greenfin horse-faced filefish (*Thamnaconus septentrionalis*) [58] and Hu male lambs (*Ovis aries*) [59]. These findings indicate that dietary protein in the PT44 group was excessively high for efficient utilization, leading to increased serum BUN concentrations in black carp. Higher levels of plasma GLU were found in the PT41 and PT44 groups, in agreement with results for snout bream (*Megalobrama amblycephala*) [60]. During protein catabolism, certain glucogenic amino acids are generated, which can be converted into glucose through deamination [61]. Collectively, the results indicate that over-intake of dietary protein in black carp can increase certain glucogenic amino acids and induce high levels of plasma GLU, while an appropriate level of dietary protein can increase protein deposition, improve ammonia–nitrogen utilization efficiency, and promote growth.

Regarding lipid metabolism, HDL-C and LDL-C are essential for cholesterol trafficking into and out of hepatocytes, thereby facilitating their subsequent utilization or clearance [62]. TG and TC are principal components of serum lipids, synthesized primarily in the liver, and their fluctuations can indicate shifts in lipid metabolism [63,64]. Our results show that increasing dietary protein levels resulted in declining trends in serum concentrations of HDL-C, LDL-C, TG, and TC in juvenile black carp, a pattern also observed in other aquatic species, such as the greenfin horse-faced filefish [57] and the obscure puffer (*Takifugu obscurus*) [65]. In addition, TG contents were also reduced with increasing levels of dietary protein, corroborating earlier studies in common carp (*Cyprinus carpio* L.) [66]. The present results demonstrate that higher dietary protein levels in black carp lower hepatic lipid accumulation by reducing TG and TC transportation. However, there were higher expression levels of *CPT1*, *CPT2*, *PPAR-α*, and *PPAR-β* in the PT38 group compared with those in the PT30 group, similar to results reported in Nile tilapia and largemouth bass [67,68]. CPTs perform a crucial role in transporting fatty acids to mitochondria and are some of the most important rate-limiting enzymes for lipolysis [69,70]. In addition, lipid catabolism and fatty acid β-oxidation are mainly modulated by the PPAR-α and PPAR-β signal pathway [71,72,73]. Thus, our results prove that adequate dietary protein intake can reduce hepatic TAG accumulation and improve fatty acid β-oxidation by activating lipolysis gene expression levels via the PPAR-α and PPAR-β signal pathway in black carp.

As the key target genes, ACC, FAS, SCD1, and ME1 play important roles in lipogenic metabolism [74]. In this research, the hepatic levels of *ACC*, *FAS*, *SCD1*, and *ME1* were reduced in fish fed a high-protein diet, which is consistent with results for common carp [66] and grass carp [75]. Given these findings, along with lower levels of body lipids observed in our results, high levels of dietary protein in black carp may suppress fatty acid and lipid synthesis by downregulating the expression of these key lipogenic genes. Furthermore, FATPs are integral membrane proteins and contribute to the transport of cellular FA into the hepatic endoplasmic reticulum (ER) to generate triacylglycerol (TAG) [76]. DGAT2 are rate-limiting enzymes involved in the TAG biosynthetic pathway [77]. Synthesized TAG can associate with APOB100 and be exported into the bloodstream through passive diffusion or lipoprotein assembly [78]. Meanwhile, as a crucial lipid droplet (LD)-coating protein, Plin2 plays a key role in LD formation and stabilization [79], and its expression levels generally correlate with intracellular TAG levels and LD density [80]. In this study, the expression levels of *FABP1*, *FATP4*, *DGAT2*, *APOB100*, and *Plin2* were considerably downregulated in fish fed high-protein diets, which is consistent with results for Hanwoo steers (*Bos taurus*) [81]. Moreover, ORO-stained hepatopancreatic sections revealed that the area of LDS accumulation and the number of hepatic LDs (observed under transmission electron microscopy) were also reduced with higher dietary protein supplementation, in agreement with earlier reports on black sea bream (*Acanthopagrus schlegelii*) [70]. In general, lipid synthesis and long-chain fatty acids are mainly regulated by the PPAR-γ and SREBP signal pathway at the transcription level [71,72,73]. The expression levels of *PPAR-γ* and *SREBP1* also decreased with dietary protein levels, paralleling observations of Nile tilapia and largemouth bass [66,68]. These findings indicate that high dietary protein intake reduces TAG accumulation in the hepatic cells by suppressing gene expression levels associated with TAG synthesis and LD formation by suppressing the PPAR-γ and SREBP1 signaling pathway.

Cholesterol metabolic homeostasis is always mediated by cholesterol synthesis, and transport is regulated by crucial genes involved in sterol-responsive enzymes, including AACS, HMGCR, CYP51, ABCG5, and NPC1L1 [82,83]. Cytoplasmic acetoacetyl-CoA can be catalyzed by AACS and HMGCR to generate cholesterol in animals [80], and CYP51 provides a critical demethylation step by catalyzing 14α-demethylation reactions during cholesterol generation [84]. Previous studies have demonstrated a positive relationship between higher serum TC contents and the transcript levels of *AACS*, *HMGCR*, and *CYP51* in largemouth bass fed with high levels of dietary starch [80]. However, our results show that the expression levels of *AACS*, *HMGCR*, *CYP51*, *ABCG5*, and *NPC1L1* were all downregulated with increasing levels of dietary protein. Combined with the serum TC contents in our results and earlier findings for largemouth bass, this indicates that cholesterol synthesis in black carp can be decreased by higher levels of dietary protein by reducing the transcription of *AACS*, *HMGCR*, and *CYP51*. In addition, ABCG5 can transport endogenous cholesterol molecules from hepatic cells to the intestine or bile lumen, while NPC1L1 can absorb cholesterol from the intestines in hepatic cells [80]. Given the functions and variations regarding *ABCG5* and *NPC1L1* in the present results, this suggests that the transport ability of cholesterol in black carp can be decreased by decreasing these two key transporters; it can then be used to maintain intracellular cholesterol homeostasis. Moreover, cholesterol can be converted into cholesteryl ester by SOAT1 for LD biogenesis and storage in hepatic cells [85,86]. Previous researchers found higher LD counts and *SOAT* expression levels in mice with type 2 diabetes [87] and largemouth bass [80]. However, lower LD counts and *SOAT1* levels were observed in our results, indicating that hepatic LD biogenesis and storage in black carp can be mediated by reducing *SOAT1* expression levels under high dietary protein conditions.

Cholesterol metabolism is always related to bile acid synthesis and transport in humans and animals [88]. Serum TBA concentrations decreased in fish that received high-protein diets, consistent with findings reported for broilers (*Gallus gallus domesticus*) [89], suggesting that increased dietary protein levels might contribute to reduced bile acid secretion in black carp. In general, CYP7A1 and CYP27A1 are the key rate-limiting enzymes that regulate bile acid synthesis by catalyzing cholesterol oxidation in hepatocytes [90]. In addition, hepatic bile acids can enter the bile canaliculi via bile salt export pump ABCB11, a bile salt transporter located on the canalicular membrane [91]. The present study found that *CYP7A1* and *CYP27A1* gene expression was notably downregulated in fish fed the high-protein diet, while there were no changes in *ABCB11*, similar to results reported for mice [92] and pearl gentian grouper (*Epinephelus lanceolatus*) [93]. These findings indicate that high protein intake can exert a global inhibitory effect on hepatic bile acid synthesis in black carp, but it did not influence transmembrane transport in the intestinal lumen. Moreover, NTCP and OATP1 may mediate the uptake of bile acids from the portal vein into hepatocytes [94], while OSTα and MRP3 may promote bile acid transmembrane transport from hepatocytes to the systemic circulation [94]. The expression levels of *NTCP*, *OATP1*, *OSTα*, and *MRP3* decreased as dietary protein levels increased, unlike results for pearl gentian grouper (*E. lanceolatus*) with high lipid intake [93] and largemouth bass with high starch intake [80]. Considering these findings and the regulatory function of bile acid transporters, our results confirmed that in black carp, high protein intake suppresses bile acid transport in the systemic circulation and reduced the uptake from the portal vein in hepatocytes. Nevertheless, additional investigations are required to clarify the molecular regulatory pathways through which varying dietary protein levels modulate bile acid metabolism.

During the continuous oxidative metabolic process for different nutrients, animals have evolved an antioxidant system to protect cells against oxidative stress induced by ROS overload. This antioxidant defense system comprises antioxidant enzymes, including SOD, CAT, GPX, GR, GST, and TXN, as well as non-enzymatic antioxidants such as GSH [25,95]. Here, the activities/levels of T-SOD, CAT, GR, GPX, T-AOC, and GSH considerably intensified in the liver and intestine of fish in the PT38 group, whereas GST activity and MDA content decreased. These results accord with previous reports on triploid rainbow trout (*Oncorhynchus mykiss*) [96] and yellow catfish (*Pelteobagrus fulvidraco*) [25]. As a key regulator, NRROS can not only reduce ROS generation but also collaborate with CAT and GPX to eliminate ROS [97]. Our results show that the transcript levels of Cu-Zn-*SOD*, *NRROS*, *GPX1*, *GR*, and *Mn-SOD* were all upregulated, while GST-a activity and MDA concentrations were reduced with increasing dietary protein supplementation, in agreement with previous observations of grass carp [98]. Considering these findings and the results of our study, the antioxidant system in black carp may be activated by adequate levels of dietary protein by heightening antioxidant enzyme activities/expression and suppressing oxidative molecule levels. In addition, GCLC and GCLM can generate cellular GSH and increase GSH contents against oxidative stress [98]. Our study shows that *GCLC* and *GCLM* transcript levels, as well as GSH contents, were all upregulated in fish groups with higher protein intake compared with the protein-deficient group, which is similar to results for grass carp [98], indicating that higher protein intake in black carp can increase GSH’s synthesizing ability by upregulating GCLC and GCLM to protect cells against oxidative stress. Meanwhile, thioredoxins and glutaredoxins in the mitochondria play an essential role in protecting cells from oxidative stress [25]. We found that high dietary protein levels elevated the transcription of *TrxR2* and *GLRXs* in black carp hepatocytes. Given the antioxidant functions of these molecules, together with our other findings, adequate or high protein intake may alleviate ROS damage and improve redox homeostasis in mitochondria in black carp by increasing thioredoxin and glutaredoxin levels. Moreover, as the key molecule in typical antioxidative signal pathways, Nrf2 can bind to Keap1 to modulate cellular redox homeostasis [25]. During the suppression of Keap1, Nrf2 can be activated and translocated into the nucleus to increase the expression of these antioxidant enzymes or functional proteins [24]. In our study, both increased *Nrf2* and decreased *Keap1* levels were observed in fish groups receiving adequate or high dietary protein, consistent with earlier results for triploid crucian carp [24] and GIFT [99]. Together with these findings, this suggests that adequate or high protein can activate the Nrf2/Keap1 signaling pathway, thereby strengthening antioxidant capacities in black carp by upregulating the transcript abundance of enzymes involved in antioxidant defense or functional proteins.

## 5. Conclusions

Overall, the present results indicate that, under the current experimental conditions, a dietary protein level of approximately 38% (PT38) could be considered a relatively suitable protein level for black carp. This level showed favorable responses in terms of serum profiles, digestive enzyme activities, growth performance, and protein synthesis-related responses, and these changes may involve the mTOR-related signaling pathway. Appropriate protein intake was also associated with reduced whole-body and hepatic lipid accumulation, accompanied by changes in the expression of genes related to lipid catabolism and metabolism in the PPAR and SREBP1 pathways. In addition, sufficient dietary protein may enhance antioxidant defenses through modulation of the Nrf2/Keap1 signaling pathway. Together with growth performance, metabolic balance, antioxidant capacity, and feed utilization efficiency, PT38 appeared to be a relatively suitable protein level under the present experimental conditions.

## Figures and Tables

**Table 1:** Composition of the diets and nutrition level (dry matter; %).

Ingredients	Composition of Diets (%)					
	PT30	PT33	PT36	PT38	PT41	PT44
Fish meal ^1^	6.00	6.00	6.00	6.00	6.00	6.00
Casein ^2^	3.00	6.00	9.00	12.00	15.00	18.00
Chicken meal ^3^	10.00	10.00	10.00	10.00	10.00	10.00
Cottonseed meal ^4^	10.00	10.00	10.00	10.00	10.00	10.00
Fermented soybean meal ^5^	16.00	16.00	16.00	16.00	16.00	16.00
Corn protein powder ^6^	9.00	9.00	9.00	9.00	9.00	9.00
Wheat flour ^7^	18.00	18.00	18.00	18.00	18.00	18.00
Fish oil ^8^	2.00	2.00	2.00	2.00	2.00	2.00
Rapeseed oil ^9^	2.00	2.00	2.00	2.00	2.00	2.00
Ca(H_2_PO_4_)_2_^10^	1.20	1.20	1.20	1.20	1.20	1.20
Mineral premix ^11^	2.20	2.20	2.20	2.20	2.20	2.20
Vitamin premix ^12^	1.10	1.10	1.10	1.10	1.10	1.10
Soybean lecithin ^13^	0.40	0.40	0.40	0.40	0.40	0.40
Choline chloride (50%) ^14^	0.40	0.40	0.40	0.40	0.40	0.40
Microcrystalline cellulose ^10^	18.70	15.70	12.70	9.70	6.70	3.70
Total (%)	100.00	100.00	100.00	100.00	100.00	100.00
Moisture	5.19	5.17	5.15	5.26	5.20	5.21
Crude protein	30.56	33.54	36.13	38.35	41.41	43.88
Crude lipid	6.43	6.68	6.23	6.33	6.51	6.45
Ash	6.16	6.14	6.23	6.25	6.27	6.31

^1^ Guangzhou Muchang import and export trading Co., Ltd., Guangzhou, Guangdong, China; ^2^ Obtained from Gansu Hualing Dairy Co., Ltd., Lanzhou, Gansu, China. Crude protein 80.56%; ^3^ Henan Zhonggu Biotechnology Co., Ltd., Hebi, Henan, China; ^4^ Xinjiang Taikun Group Co., Ltd., Changji, Xinjiang, China; crude protein: 64%. ^5^ Golden Glory Food Co., Ltd., Ningbo, Zhejang, China; ^6^ Jiaxing Xinxin Food Tech Co., Ltd. Jiaxing, Zhengjiang, China. ^7^ Yihai Kerry Food Marketing Co., Ltd., Shanghai, China; ^8^ Qingdao Surgreen Marine Bio-Feed Co., Ltd., Qingdao, Shandong, China; ^9^ Yihai Kerry Golden Dragon Fish Food Group Co., Ltd., Shanghai, China; ^10^ Sinopharm Chemical Reagent Co., Ltd., Shanghai, China; ^11^ KI 0.4 mg, CoCl_2_·6H_2_O 200 mg, CuSO_4_·5H_2_O 16 mg, FeSO_4_·7H_2_O 300 mg, ZnSO_4_·H_2_O 15 mg, MnSO_4_·H_2_O 45 mg, MgSO_4_·7H_2_O 20 mg, NaCl 0.6 mg; ^12^ Vitamin A 20 mg, Vitamin D3 3 mg, Vitamin C 300 mg, Vitamin E 300 mg, thiamin 20 mg, riboflavin 10 mg, pyridoxine 20 mg, Vitamin B12 0.2 mg, Vitamin K3 5 mg, inositol 1000 mg, pantothenic acid 30 mg, folic acid 3 mg, niacin acid 50 mg, biotin 1 mg; ^13^ Jiangsu Yuanshengyuan Biological Engineering Co., Ltd., Nanjing, Jiangsu, China; ^14^ Zhejiang Yixing Feed Group Co., Ltd., Jiaxing, Zhejiang, China. PT30, PT33, PT36, PT38, PT41, and PT44 represent the dietary protein levels of 30%, 33%, 36%, 38%, 41%, and 44%, respectively.

**Table 2:** Amino acid composition of the six experimental diets.

Amino Acids (Dry Matter; %)	Groups					
	PT30	PT33	PT36	PT38	PT41	PT44
Essential amino acids						
Threonine	1.10	1.21	1.32	1.40	1.46	1.59
Valine	1.49	1.65	1.91	2.02	2.12	2.37
Methionine	0.25	0.37	0.49	0.52	0.59	0.64
Isoleucine	2.64	2.95	3.17	3.38	3.47	3.87
Leucine	2.64	2.85	3.17	3.38	3.47	3.87
Phenylalanine	1.57	1.67	1.84	1.95	2.05	2.25
Lysine	1.55	1.91	2.06	2.19	2.27	2.57
Histidine	0.75	0.77	0.97	0.97	1.02	1.10
Arginine	1.77	1.85	1.94	2.00	1.95	2.01
Non-essential amino acids						
Aspartic acid	2.60	2.85	3.01	3.19	3.27	3.55
Serine	1.29	1.37	1.53	1.61	1.69	1.86
Glutamic acid	6.15	6.95	7.41	7.92	8.25	9.08
Glycine	1.46	1.50	1.59	1.61	1.61	1.72
Alanine	1.75	1.80	1.98	2.01	2.04	2.17
Cysteine	0.47	0.48	0.52	0.44	0.45	0.55
Proline	1.92	2.12	2.53	2.72	2.92	3.24
Tyrosine	1.06	1.12	1.38	1.54	1.58	1.75
∑EAA ^1^	13.77	15.24	16.85	17.82	18.40	20.27
∑NEAA ^2^	16.71	18.19	19.95	21.05	21.80	23.91
∑TAA ^3^	30.48	33.42	36.80	38.87	40.20	44.18

^1^ ∑EAA, essential amino acids; ^2^ ∑NEAA, non-essential amino acids; ^3^ ∑TAA, total amino acids. PT30, PT33, PT36, PT38, PT41, and PT44 represent the dietary protein levels of 30%, 33%, 36%, 38%, 41%, and 44%, respectively.

**Table 3:** Effects of dietary protein level on growth and body composition of black carp (*Mylopharyngodon piceus*).

Items	Groups						
	PT30	PT33	PT36	PT38	PT41	PT44	ANOVA (*p* Value)
Growth performance							
IBW (g) ^1^	10.81 ± 0.12	10.87 ± 0.08	10.71 ± 0.22	10.80 ± 0.27	10.73 ± 0.22	11.06 ± 0.25	0.38
FBW (g) ^2^	29.09 ± 0.51 ^a^	31.74 ± 0.61 ^ab^	33.93 ± 0.89 ^bc^	36.09 ± 1.49 ^c^	35.99 ± 0.22 ^c^	36.59 ± 2.58 ^c^	0.01
WG (%) ^3^	172.00 ± 4.13 ^a^	191.95 ± 6.11 ^ab^	216.81 ± 5.87 ^bc^	234.34 ± 17.93 ^c^	235.45 ± 8.10 ^c^	230.47 ± 16.16 ^c^	0.01
SGR (%) ^4^	1.79 ± 0.03 ^a^	1.91 ± 0.04 ^ab^	2.06 ± 0.04 ^bc^	2.15 ± 0.09 ^c^	2.16 ± 0.04 ^c^	2.14 ± 0.09 ^c^	0.01
VSI (%) ^5^	6.35 ± 0.09 ^c^	6.28 ± 0.07 ^bc^	6.16 ± 0.06 ^ab^	6.08 ± 0.08 ^a^	6.05 ± 0.05 ^a^	6.01 ± 0.12 ^a^	0.01
HSI (%) ^6^	1.54 ± 0.09 ^b^	1.43 ± 0.03 ^ab^	1.39 ± 0.05 ^a^	1.40 ± 0.04 ^a^	1.38 ± 0.04 ^a^	1.32 ± 0.09 ^a^	0.02
ISI (%) ^7^	1.26 ± 0.07 ^c^	1.26 ± 0.01 ^c^	1.19 ± 0.02 ^c^	1.16 ± 0.06 ^bc^	1.07 ± 0.05 ^ab^	1.01 ± 0.09 ^a^	0.01
CF (g/cm^3^) ^8^	1.66 ± 0.01	1.69 ± 0.01	1.67 ± 0.02	1.68 ± 0.02	1.67 ± 0.01	1.69 ± 0.06	0.73
FCR ^9^	1.92 ± 0.01 ^e^	1.80 ± 0.01 ^d^	1.69 ± 0.01 ^c^	1.53 ± 0.03 ^b^	1.51 ± 0.01 ^b^	1.46 ± 0.02 ^a^	0.01
Body composition (%)							
Moisture	71.09 ± 0.30 ^ab^	71.90 ± 0.76 ^b^	70.82 ± 0.52 ^a^	70.75 ± 0.26 ^a^	71.15 ± 0.40 ^ab^	71.02 ± 0.44 ^ab^	0.40
Crude protein	15.41 ± 0.09 ^a^	16.22 ± 0.11 ^b^	16.28 ± 0.06 ^b^	16.66 ± 0.15 ^c^	16.67 ± 0.05 ^c^	16.74 ± 0.04 ^c^	0.01
Crude lipid	10.38 ± 0.37 ^c^	10.20 ± 0.03 ^c^	10.07 ± 0.03 ^bc^	9.80 ± 0.14 ^ab^	9.70 ± 0.17 ^ab^	9.51 ± 0.25 ^a^	0.02
Ash	2.53 ± 0.03 ^a^	2.58 ± 0.03 ^ab^	2.64 ± 0.06 ^abc^	2.66 ± 0.08 ^bc^	2.72 ± 0.09 ^c^	2.71 ± 0.06 ^c^	0.02

^1^ IBW, initial body weight; ^2^ FBW, final body weight; ^3^ WG, Weight gain; ^4^ SGR, Specific growth rate; ^5^ VSI, Viscerosomatic index; ^6^ HSI, Hepatosomatic index; ^7^ ISI, Intestinal somatic index; ^8^ CF, Condition factor; ^9^ FCR, Feed conversion ratio. Values are means ± SD (For each treatment, n = 3 biological replicates). Values are expressed as mean ± SD (n = 3 replicate tanks per treatment, with 15 fish in each tank). Different lowercase superscript letters within the same row indicate significant differences among groups according to one-way ANOVA followed by Duncan’s multiple range test (*p* < 0.05); values without lowercase superscript letters indicate no significant differences among groups. PT30, PT33, PT36, PT38, PT41, and PT44 represent the dietary protein levels of 30%, 33%, 36%, 38%, 41%, and 44%, respectively.

**Table 4:** Effects of dietary protein level on serum biochemical parameters in black carp (*Mylopharyngodon piceus*).

Items	Groups						
	PT30	PT33	PT36	PT38	PT41	PT44	ANOVA (*p* Value)
HDL-C (mmol/L) ^1^	0.40 ± 0.02 ^c^	0.39 ± 0.02 ^bc^	0.37 ± 0.03 ^abc^	0.36 ± 0.01 ^ab^	0.34 ± 0.01 ^a^	0.35 ± 0.02 ^a^	0.01
LDL-C (mmol/L) ^2^	1.73 ± 0.02 ^c^	1.71 ± 0.02 ^c^	1.59 ± 0.02 ^b^	1.54 ± 0.01 ^a^	1.52 ± 0.01 ^a^	1.52 ± 0.02 ^a^	0.01
TG (mmol/L) ^3^	4.94 ± 0.06 ^e^	3.71 ± 0.09 ^d^	3.63 ± 0.02 ^cd^	3.59 ± 0.08 ^c^	3.47 ± 0.03 ^b^	3.29 ± 0.08 ^a^	0.01
TC (mmol/L) ^4^	4.06 ± 0.09 ^d^	3.96 ± 0.05 ^c^	3.77 ± 0.02 ^b^	3.75 ± 0.03 ^b^	3.66 ± 0.02 ^a^	3.64 ± 0.01 ^a^	0.01
AST (U/L) ^5^	154.43 ± 26.25 ^a^	176.07 ± 11.69 ^ab^	184.63 ± 13.99 ^ab^	189.93 ± 15.26 ^ab^	207.57 ± 17.32 ^b^	212.27 ± 27.25 ^b^	0.03
ALT (U/L) ^6^	4.50 ± 1.85	6.20 ± 2.40	6.77 ± 0.29	6.47 ± 0.55	5.80 ± 1.73	5.87 ± 1.35	0.58
ALP (U/L) ^7^	129.33 ± 6.80 ^a^	138.85 ± 6.05 ^b^	140.90 ± 4.22 ^b^	187.86 ± 5.81 ^d^	175.87 ± 5.15 ^c^	167.55 ± 2.75 ^c^	0.01
GLU (mmol/L) ^8^	3.50 ± 0.18 ^a^	3.63 ± 0.13 ^a^	4.01 ± 0.07 ^b^	4.27 ± 0.06 ^c^	4.72 ± 0.02 ^d^	4.79 ± 0.07 ^d^	0.01
TBA (umol/L) ^9^	7.53 ± 0.42 ^c^	8.45 ± 0.45 ^d^	7.25 ± 0.25 ^c^	6.67 ± 0.21 ^b^	5.87 ± 0.06 ^a^	5.80 ± 0.30 ^a^	0.01
ALB (g/L) ^10^	8.45 ± 0.05 ^ab^	8.53 ± 0.21 ^ab^	8.70 ± 0.10 ^bc^	8.93 ± 0.21 ^c^	8.67 ± 0.06 ^b^	8.35 ± 0.05 ^a^	0.01
BUN (mmol/L) ^11^	2.20 ± 0.05 ^a^	2.23 ± 0.09 ^a^	2.31 ± 0.07 ^a^	2.51 ± 0.04 ^b^	2.61 ± 0.07 ^b^	2.62 ± 0.01 ^b^	0.01

^1^ HDL-C, high-density lipoprotein cholesterol; ^2^ LDL-C, low-density lipoprotein cholesterol; ^3^ TG, triglyceride, ^4^ TC, total cholesterol; ^5^ AST, aspartate aminotransferase; ^6^ ALT, alanine aminotransferase; ^7^ ALP, alkaline phosphatase; ^8^ GLU, glucose; ^9^ TBA, total bile acids; ^10^ ALB, albumin; ^11^ BUN, blood urea nitrogen; Values are expressed as mean ± SD. The value of n = 3 represents biological replicates, with each biological replicate consisting of pooled samples from 15 fish within one replicate tank. Different lowercase superscript letters within the same row indicate significant differences among groups according to one-way ANOVA followed by Duncan’s multiple range test (*p* < 0.05); values without lowercase superscript letters indicate no significant differences among groups. PT30, PT33, PT36, PT38, PT41, and PT44 represent dietary protein levels of 30%, 33%, 36%, 38%, 41%, and 44%, respectively.

**Table 5:** Effects of dietary protein level on digestive enzymes in the hepatopancreas and intestine of black carp (*Mylopharyngodon piceus*).

Items	Groups						
	PT30	PT33	PT36	PT38	PT41	PT44	ANOVA (*p* Value)
Hepatopancreas							
AMS (U/g prot) ^1^	228.84 ± 14.95 ^ab^	230.59 ± 15.78 ^ab^	245.83 ± 6.85 ^ab^	249.68 ± 13.25 ^b^	235.75 ± 16.63 ^ab^	226.85 ± 11.34 ^a^	0.14
TRY (U/mg prot) ^2^	467.84 ± 24.84 ^a^	455.42 ± 9.56 ^a^	604.46 ± 9.56 ^c^	666.57 ± 15.86 ^d^	575.48 ± 15.86 ^bc^	554.78 ± 21.38 ^b^	0.01
LPS (umol/min/mg prot) ^3^	0.22 ± 0.01 ^a^	0.24 ± 0.01 ^a^	0.29 ± 0.01 ^b^	0.34 ± 0.01 ^c^	0.30 ± 0.01 ^b^	0.28 ± 0.01 ^b^	0.01
Intestine							
AMS (U/g prot) ^1^	124.52 ± 5.68 ^a^	134.08 ± 11.09 ^abc^	140.92 ± 10.16 ^bc^	147.32 ± 10.02 ^bc^	140.82 ± 8.72 ^c^	131.23 ± 8.65 ^ab^	0.03
TRY (U/mg prot) ^2^	124.03 ± 3.62 ^a^	136.22 ± 5.57 ^b^	180.51 ± 4.24 ^d^	189.37 ± 7.57 ^e^	170.44 ± 2.69 ^c^	162.80 ± 6.64 ^c^	0.01
LPS (umol/min/mg prot) ^3^	0.33 ± 0.02 ^a^	0.36 ± 0.02 ^b^	0.42 ± 0.02 ^cd^	0.44 ± 0.01 ^d^	0.42 ± 0.01 ^cd^	0.39 ± 0.01 ^c^	0.01

^1^ AMS, amylase; ^2^ TRY, trypsin; ^3^ LPS, lipase. Values are means ± SD (For each treatment, n = 3 biological replicates). Mean values with different superscripts in the same row differ significantly based on Duncan’s test (*p* < 0.05). Values are expressed as mean ± SD. The value of n = 3 represents biological replicates, with each biological replicate consisting of pooled samples from 15 fish within one replicate tank. Different lowercase superscript letters within the same row indicate significant differences among groups according to one-way ANOVA followed by Duncan’s multiple range test (*p* < 0.05); values without lowercase superscript letters indicate no significant differences among groups. PT30, PT33, PT36, PT38, PT41, and PT44 represent dietary protein levels of 30%, 33%, 36%, 38%, 41%, and 44%, respectively.

**Table 6:** Effects of dietary protein level on the antioxidative and oxidative indices in the hepatopancreas and intestine of black carp (*Mylopharyngodon piceus*).

Items	Groups						
	PT30	PT33	PT36	PT38	PT41	PT44	ANOVA (*p* Value)
Hepatopancreas							
T-SOD (U/mg prot) ^1^	11.81 ± 0.89 ^a^	15.52 ± 0.91 ^b^	20.93 ± 2.27 ^c^	28.67 ± 1.31 ^e^	27.27 ± 0.77 ^de^	26.66 ± 0.68 ^d^	0.01
CAT (U/mg prot) ^2^	58.05 ± 1.64 ^a^	62.28 ± 2.67 ^b^	66.41 ± 1.81 ^c^	71.67 ± 1.67 ^d^	67.63 ± 2.04 ^c^	66.26 ± 2.86 ^c^	0.01
GPX (U/mg prot) ^3^	130.82 ± 6.05 ^a^	145.39 ± 5.86 ^b^	164.71 ± 10.37 ^c^	204.40 ± 14.64 ^e^	196.65 ± 6.47 ^de^	189.20 ± 3.35 ^d^	0.01
GST (U/mg prot) ^4^	186.65 ± 10.56 ^b^	181.04 ± 8.42 ^b^	178.93 ± 19.02 ^b^	168.49 ± 53.6 ^ab^	153.55 ± 20.10 ^ab^	131.30 ± 8.10 ^a^	0.05
GSH (μmol/g prot) ^5^	163.88 ± 3.12 ^a^	186.04 ± 1.77 ^b^	272.67 ± 3.14 ^c^	315.45 ± 4.32 ^e^	298.42 ± 4.31 ^d^	257.01 ± 5.48 ^c^	0.01
T-AOC (mmol/g prot) ^6^	2.55 ± 0.08 ^b^	2.56 ± 0.10 ^b^	2.61 ± 0.07 ^b^	2.70 ± 0.16 ^b^	2.30 ± 0.08 ^a^	2.28 ± 0.03 ^a^	0.01
MDA (mmol/g prot) ^7^	64.00 ± 2.55 ^d^	63.69 ± 3.12 ^d^	58.77 ± 1.03 ^c^	56.16 ± 1.44 ^c^	48.82 ± 1.05 ^b^	44.10 ± 1.47 ^a^	0.01
Intestine							
T-SOD (U/mg prot) ^1^	7.11 ± 0.52 ^ab^	7.67 ± 0.58 ^b^	8.47 ± 0.28 ^c^	9.77 ± 0.16 ^d^	7.11 ± 0.52 ^ab^	6.75 ± 0.54 ^a^	0.01
CAT (U/mg prot) ^2^	45.70 ± 2.66 ^ab^	49.48 ± 6.11 ^b^	58.05 ± 1.64 ^c^	63.69 ± 3.12 ^d^	56.16 ± 1.44 ^c^	44.00 ± 1.49 ^a^	0.01
GPX (U/mg prot) ^3^	136.44 ± 7.97 ^a^	149.24 ± 4.98 ^ab^	169.40 ± 10.28 ^c^	184.31 ± 12.39 ^d^	155.71 ± 13.39 ^bc^	140.44 ± 4.32 ^a^	0.000
GST (U/mg prot) ^4^	84.17 ± 2.20 ^d^	84.09 ± 3.83 ^d^	81.35 ± 4.96 ^bc^	78.1 ± 5.09 a^bc^	76.01 ± 3.88 ^ab^	74.11 ± 3.41 ^a^	0.01
GSH (μmol/g prot) ^5^	170.92 ± 3.19 ^a^	223.63 ± 2.51 ^b^	268.11 ± 6.02 ^d^	283.59 ± 7.02 ^e^	278.67 ± 6.79 ^de^	254.50 ± 13.24 ^c^	0.01
T-AOC (mmol/g prot) ^6^	0.60 ± 0.05 ^a^	0.75 ± 0.08 ^b^	0.84 ± 0.17 ^bc^	1.28 ± 0.06 ^d^	0.92 ± 0.07 ^c^	0.81 ± 0.06 ^bc^	0.01
MDA (mmol/g prot) ^7^	5.85 ± 0.80 ^b^	4.15 ± 0.92 ^a^	3.85 ± 0.59 ^a^	3.85 ± 0.59 ^a^	3.49 ± 0.58 ^a^	3.38 ± 0.31 ^a^	0.01

^1^ T-SOD, total superoxide dismutase; ^2^ CAT, catalase; ^3^ GPx, glutathione peroxidase; ^4^ GST, glutathione S-transferase; ^5^ GSH, glutathione; ^6^ T-AOC, total antioxidant capacity; ^7^ MDA, malondialdehyde; Values are expressed as mean ± SD. The value of n = 3 represents biological replicates, with each biological replicate consisting of pooled samples from 15 fish within one replicate tank. Different lowercase superscript letters within the same row indicate significant differences among groups according to one-way ANOVA followed by Duncan’s multiple range test (*p* < 0.05); values without lowercase superscript letters indicate no significant differences among groups. PT30, PT33, PT36, PT38, PT41, and PT44 represent dietary protein levels of 30%, 33%, 36%, 38%, 41%, and 44%, respectively.

## Data Availability

The data presented in this study are available in the main article.

## Supplementary Materials

The following supporting information can be downloaded at: https://www.mdpi.com/article/10.3390/metabo16060391/s1, Table S1. The abbreviations are used in this manuscript. Table S2. Primers used in this study.
