# Supplementary material for: Effects of Dietary Protein Levels on Growth, Serum Physiology, Protein and Lipid Metabolism, and Antioxidant Responses in Black Carp (Mylopharyngodon piceus)
Source: Metabolites. 2026 Jun 4;16(6):391. doi: 10.3390/metabo16060391 (PMC13303830; doi:10.3390/metabo16060391)
Supplement: Supplementary file 1 [file metabolites-16-00391-s001.zip › metabolites-4330862-supplementary.pdf]

Supplementary Files

Table S1. The abbreviations are used in this manuscript.

SGR	Specific growth rate
FCR	Feed conversion ratio
HDL-C	High-density lipoprotein cholesterol
LDL-C	Low-density lipoprotein cholesterol
TC	Total cholesterol
TG	Triglyceride
ALT	Alanine aminotransferase
AST	Aspartate aminotransferase
TBA	Total bile acids
GLU	Glucose
ALP	Alkaline phosphatase
ALB	Albumin
AMS	α-Amylase
TRY	Trypsin
LPS	Lipase
mTOR	mechanistic target of rapamycin
S6K1	Ribosomal protein S6 kinase 1
4E-BP	Eukaryotic translation initiation factor 4E-binding protein
IGF-I	Insulin-like growth factor I
IGF-II	Insulin-like growth factor II
AKT	Protein kinase B
CPT1α	Carnitine palmitoyl transferase 1 Alpha
CPT2	Carnitine Palmitoyltransferase 2
PPARα	Peroxisome proliferator-activated receptor alpha
PPARδ	Peroxisome proliferator-activated receptor delta
PPARγ	Peroxisome proliferator-activated receptor gamma
PPARs	Peroxisome proliferator-activated receptors
SREBP1	Sterol regulatory element-binding protein 1
SREBPs	Sterol regulatory element-binding proteins
ACC	Acetyl-CoA carboxylase
FAS	Fatty acid synthase
SCD	Stearoyl-CoA desaturase
ME1	Malic enzyme 1
FABP-L	Fatty acid-binding protein liver type
FATP4	Fatty Acid transport protein 4
DGAT2	Diacylglycerol O-acyltransferase 2
Plin2	Perilipin 2
APOB100	Apolipoprotein B-100
AACS	Acetoacetyl-CoA synthetase
HMGCR	3-Hydroxy-3-Methylglutaryl-CoA reductase
CYP51	Lanosterol 14α-demethylase
SOAT1	Sterol O-Acyltransferase 1
ABCG5	ATP-binding cassette subfamily g member 5
NPC1L1	Niemann-pick c1-like 1
CYP7A1	Cholesterol 7α-hydroxylase
CYP27A1	Sterol 27-hydroxylase
ABCB11	ATP-binding cassette subfamily B member 11
NTCP	Na^+/Taurocholate cotransporting polypeptide
OATP1	Organic anion transporting polypeptide 1
OSTα	Organic solute transporter alpha
MRP3	Multidrug resistance-associated protein 3
Cu/Zn-SOD	Cu/Zn superoxide dismutase

Mn-SOD	Manganese superoxide dismutase
CAT	Catalase
GPX1	Glutathione peroxidase 1
GR	Glutathione reductase
GCLC	Glutamate-cysteine ligase catalytic subunit
GCLM	Glutamate-cysteine ligase modifier subunit
GSTA	Glutathione s-transferase alpha
NRROS	Negative regulator of reactive oxygen species
Nrf2	Nuclear factor erythroid 2–related factor 2
Keap1a	Kelch-Like ECH-associated protein 1a
Keap1b	Kelch-Like ECH-associated protein 1b
TrxR2	Thioredoxin reductase 2
GLRX1	Glutaredoxin 1
GLRX2	Glutaredoxin 2
GLRX3	Glutaredoxin 3
T-AOC	total antioxidant capacity
GST	glutathione s-transferase

Table S2. Primers used in this study.

Gene	Forward (5′-3′)	Reverse (5′-3′)
IGF-1	GTTGACTCCTGCGACACTG	CGTCTCGAACTAGGTCCATAT
IGF-2	GCAGGCTCTTCACAAGGACA	GCAAGCAGGATGGATGGAAC
mTOR	TCAGCCTTCAGGGTATCAA	GCTTGCGTGTCGTAGTTTT
S6K1	GTGCCTCATCTCGGCTTGG	GGCTTGAATGGTGGTTCTACT
4EBP1	ACACGCAGCAGAGCAAGAG	TGTCATAAATGATCCGAGGTCC
AKT	CCATTCAAACCGCAAGTGT	CGAACGGCTCCATACCAT
CPT1α	GAAGATTCGGCTTTCCTCAA	CCCTCCGCTGGTGACATA
CPT2	ATGCCGCCATAAACCACAA	GCCATAGCCCACTCCGAAA
PPARα	GTCAATACTGCCGCTTCCG	GCTTTCAGCCTCTGCTTCTCA
PPARδ	GCCAAACGGGTCAACAACG	CCTGCCACAGCGAGTCCAT
PPARγ	CAGACTCGCTCCAGCTCTTCG	TGTAAGCACCAGTCCACCTCC
SREBP1	TCTGGAGACACCGCAAGCA	GGATGGACCACAACATAGCACA
ACC	CCCCTCTACACCAACCTTT	ATGTCGCTGTCCGTCTTTA
FAS	TGGACGGGTAACTGGGTGA	CAGAACGGATGCGAGTGGG
SCD	CCCTTCAGCATCTCCTTT	GTGGTAATGTGGCCTTGTA
ME1	TCGCTAAGGAGGAGTGTTTG	GTTTCTTGATCTGTGGGTGC
FABP-L	GGTCAAGTCGGTGGTTCA	ATGCGTTTGCTTGTCCTC
FATP4	GATTCTACCGTTCATCTACCC	GAATGATCCTGCCGACTA
DGAT2	ATCTATGATTGGCAAACACCC	CAGCAGTCTTTACCAGCTTCAC
Plin2	GAGTGGACACGGCCCTAA	GAACCCAGGCGGACATAG
APOB100	GATTGTAAAGTTTGAGGCTGAC	TGCATGAATTTCGTAGGG
AACS	GCTCGGCTCAACTACGCT	CGAACAACGCCACATCTT
HMGCR	CCTGGACGACTTTGGCTAT	CCTGTGAAGGGTCCTGTCT
CYP51	TCGCCTCAGACTGTAGCAG	GAGGTAGCGGTCAGGGTT
SOAT1	TGGCGTTCTTTGCCTTTC	ATTCCATGTGCGGTAATAGTTAG
ABCG5	CTTGGCAATGGGCTTCGG	GCTCTGTCGCTTGTCGTTC
NPC1L1	ATCATAGTCTTTGCCTGCGTCTT	TCTTGCCTTTCCGAGCCC
CYP7A1	GGCTGTGCTCTTCAGTTTGG	CGGATGACGGCATGGTAG
CYP27A1	ATTTGGCTTCGGCATTCG	TCGGCTTCACAGTCCTTCC
ABCB11	CATTCGTGGTGGGCTTCC	GCTCCATTCCCGTCAGTTTT
NTCP	CTGTAGGAGGAAATGAGGCG	CGAGAAGATGGAGGTGAACG
OATP1	TCACAAGAAATGCGGTGGAAG	AGCACGCACAGGAAGAAGGA
OSTα	TAAGAAAGTACCGGCAAATAAG	ACTGCAAAGTATGTGGCTGA
MRP3	TCGGAGACCATCACGGGCACT	AACCACCTGTTGGAGACAATACC
Cu/Zn-SOD	GCAGGTCCGCACTTCAA	GACAGGGACAGCATTTGGT
Mn-SOD	GCAGGGCACTACAGGTCTC	CTCCCAGTTCACAACATTCC

CAT	CAGTATCTTACGTGATGGGTCT	GGAAGTTGCCGTTGGAGAT
GPX1	GCAACCAGTTCGGACATCA	GCGTTCTCACCGTTCACTT
GR	CAGAACACTACACGTCCAGG	CAAACAGTCGGTGAGCAAG
GCLC	CGGCATCCTTCAGTTCCTC	TCTTTCATCCATTTCCACCA
GCLM	TTCGTTAGGCGTATCTCAG	TCTCCAACTCTTCCCACAG
NRROS	AACTTCTGGACTTGAGGGAT	TGTACCAGTCTAAATGGCAAC
GSTA	TTGGAGAAGATGGGTAAAGGG	TAGGACAGTGAAGTCGTGGG
Nrf2	GAAGCGGCAGCAGCATTT	CTGTGCGATTTCTCCATTTGTT
Keap la	ACGGGCTGCTCTGTGATCTGG	ACGGGCTGCTCTGTGATCTGG
Keap lb	CGCCATCGGCATCGCCAACT	TCCTGCGTAGCCACCTGACTGAA
TrxR2	TGTAAATGCCAAAGGGAAAGA	CACTTGTGATCCCGAACTCC
GLRX1	TTGAAGCCGTCCTGTCCT	GCGCCTGTGATCTGTTGA
GLRX2	AAATGACAGGTGCCAGAAC	CAAACAGCATGGACGACA
GLRX3	AGAGGCAGTCCCGGAGGT	CCCAGCCGCTGCACCTTA
β-actin	CCTTCTTGGGTATGGAGTC	GTCAGCAATGCCAGGGTA
